# Determination of Suitable Microspore Stage and Callus Induction from Anthers of Kenaf (*Hibiscus cannabinus* L.)

**DOI:** 10.1155/2014/284342

**Published:** 2014-03-13

**Authors:** Ahmed Mahmood Ibrahim, Fatimah Binti Kayat, Zeti Ermiena Surya Mat Hussin, Dwi Susanto, Mohammed Ariffulah

**Affiliations:** Faculty of Base Industry (FIAT), Universiti Malaysia Kelantan, Jeli campus, Locked Bag 100, 17600 Jeli, Kelantan, Malaysia

## Abstract

Kenaf (*Hibiscus cannabinus* L.) is one of the important species of *Hibiscus* cultivated for fiber. Availability of homozygous parent lines is prerequisite to the use of the heterosis effect reproducible in hybrid breeding. The production of haploid plants by anther culture followed by chromosome doubling can be achieved in short period compared with inbred lines by conventional method that requires self pollination of parent material. In this research, the effects of the microspore developmental stage, time of flower collection, various pretreatments, different combinations of hormones, and culture condition on anther culture of KB6 variety of Kenaf were studied. Young flower buds with immature anthers at the appropriate stage of microspore development were sterilized and the anthers were carefully dissected from the flower buds and subjected to various pretreatments and different combinations of hormones like NAA, 2,4-D, Kinetin, BAP, and TDZ to induce callus. The best microspore development stage of the flower buds was about 6–8 mm long collected 1-2 weeks after flower initiation. At that stage, the microspores were at the uninucleate stage which was suitable for culture. The best callus induction frequency was 90% in the optimized semisolid MS medium fortified with 3.0 mg/L BAP + 3.0 mg/L NAA.

## 1. Introduction

Kenaf (*Hibiscus cannabinus* L.) belongs to the Malvaceae family and section Furcaria. It is closely related to cotton, okra, hollyhock, and roselle. Nowadays, it has been cultivated in 20 countries worldwide and its total production (kenaf and allied crops) is 352,000 tons (2010/2011). Kenaf is an annual fiber crop cultivated for numerous uses (paper pulp, fabrics, textile, building materials, biocomposites, bedding material, oil absorbents, etc.) [1]. In Malaysia, this plant is considered new and cultivated to replace tobacco plantation that has been no longer supported by the government [2]. Kenaf grows quickly and will achieve 5 to 6 m in height and 2.5 to 3.5 cm in diameter within 5 to 6 months. Kenaf has a unique combination of long bast and short core fibers which makes it suitable for a range of paper and cardboard products. Fifty-five percent of dried kenaf stalks are used to make paper. Waste products from the process can be made into fertilizer and feed binder. When kenaf is grown in home gardens for fiber, the more tender upper leaves and shoots are sometimes eaten either raw or cooked [3]. Alternative uses, the top leafy portion of the kenaf plant is not useful for pulping. Therefore, this part of the plant would be useful as forage if harvest equipment could be practically adapted to a dual collection operation. One of the most popular methods for production of haploids is through anther and microspore culture on the artificial culture medium. Haploids are plants (sporophytes) that contain a gametic chromosome number (n) as in the egg and pollen cell. Each pollen mother cell (PMC) in the anther produces 4 microspore or pollen grains. Thus, anther with microspore or isolated pollen grains can be cultured on artificial medium to raise haploid plant. Haploid production through anther culture has been referred to as androgenesis [4].

The first time haploid plants were discovered in* Datura stramonium* by A.D Bergner in 1921. After the initial reports of successful production of haploids from anther culture in Datura [5], haploids have been obtained in more than 150 species belonging to 23 families of angiosperms [6]. Many parameters have been recognized as particularly important for successful anther and microscope culture such as conditions of growth of donor plant, genotype of donor plant, cold and hot pretreatment to anthers, the developmental stage of anther/microspore, and the culture medium and the conditions during culture growth. Anther and ovule culture have advantage of production of haploid plants and production of homozygous diploid lines through chromosome doubling, thus reducing the time required to produce homozygous lines.

The first step in hybrid seed development is to develop inbred parent lines by repeated self-pollination. This can be a very slow process which requires 6-7 generation. However, by culturing pollen grains in the laboratory, haploid plants that contain only one copy of each chromosome can be produced. These plants can then be induced to double their chromosome number by a chemical treatment, quickly resulting in plants that have two identical sets of chromosomes, or are completely homozygous. This procedure can dramatically reduce the time required to develop inbred parents for the production of F1 hybrid varieties. Apparently, haploid and double haploid shoot production occur as a result of organogenesis from an initial proliferation of callus and not via androgenesis [7].* In vitro* androgenesis has been the mainstream approach in the production of pure lines via haploids which is later doubled to restore the diploidy [8]. A direct and simple regeneration procedure using the kenaf shoot apex was reported [9]. Production of callus and its subsequent regeneration is the prime steps of crop plant to be manipulated by biotechnological means [10]. First described plant regeneration from two-year-old callus was of* Gossypium hirsutum* L. cv Coker 310 via somatic embryogenesis as reported by Davidonis and Hamilton [11]. Since then, significant progress has been reported in cotton tissue culture [12].

Even though* in vitro* shoot regeneration has been reported [13, 14], there are no reports on anther culture in kenaf. Hence, the aim of this study was to determine the best microspore development stage of the flower buds in KB6 variety of kenaf and cultured on MS medium with different concentrations of hormones for the induction of callus.

## 2. Materials and Methodology

### 2.1. Plant Material & Determination of Microspore Development Stage

**Figure 1:**
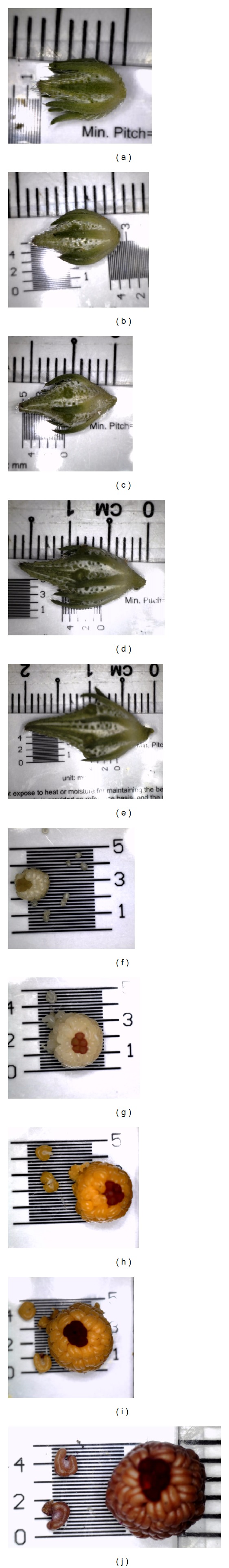
(a)–(e) Different size of flower buds, (f)–(j) anther length and diameter of anther bunch.

**Figure 2:**
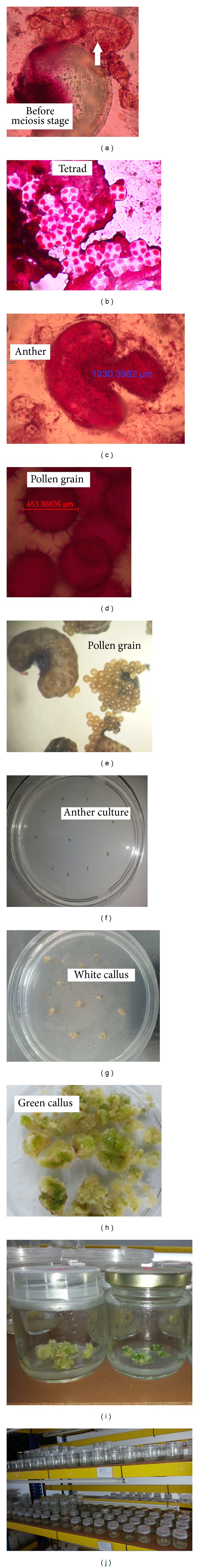
(a) Anther before meiosis stage, (b) Tetrad stage, (c) anther length, (d) pollen grain length, (e) mature anther and pollen grain, (f) anther culture in petri dish contain callus media, (g) white callus induction after 8 weeks (first subculture), (h) green callus after 6 weeks (second subculture), (i) and (j) callus in bottle.

Seeds of kenaf KB6 were used as anther donors after 8 weeks from seeds plantation. Flower buds of different lengths 6, 8, 12, 16, and 20 mm (Table 1, Figure 1) in different time after the date of the flower bud initiation (Table 2) were collected for anther culture having the different microspore and pollen grain stages (Figures 2(a)–2(d)). Five buds from each length were collected and carried into the laboratory immediately. After the cold pretreatment and prior to culturing, the stage of microspores was checked under microscope. This was performed by removing one or two anthers then stained with 4% acetocarmine (W/V) in 50% (v/v) acetic acid [15]. The lengths of flower buds, anther length, and anther bunch diameters were measured by the digital microscope.

### 2.2. Pretreatment & Sterilization

First, the buds were washed with sterile distilled water for 5 min to remove the dust and disinfected for 45 second in 70% alcohol and 8–10 min in 3% sodium hypochlorite (NaCLO) with 2-3 drops tween 20 [16]. The buds were rinsed 4 times with double sterile distilled water for three minutes each time and dried on sterile filter paper and kept the sterile bottles in refrigerator under 4–6° Celsius for chilling pretreatment for 3, 6, 9, 12 and 15 days [17, 18]. The buds were carefully teased and anthers were removed. The filaments were cut from anthers to avoid* in vitro* callusing from cut places.

### 2.3. Culture Medium

Suitable anthers were inoculated on the solid medium (8% agar) that contained four types of plant growth regulator (PGR): two types of Auxins NAA and 2,4-D and two types of cytokinins, BAP and Kinetin (Table 3). Each type of media consisted of six Petri dishes where 10 anthers have been inculcated (contained 25 mL of MS medium) (Figure 2(f)). The pH was adjusted to 5.7 and incubated at 25°C in darkness place. After six weeks, the induced calli were transferred to new media which contain 0.5 mg/L TDZ (thidiazuron) with 0.5 mg/L NAA at 24–27°C and were exposed to light at an intensity of about 2000 Lux for 16 hours per day [19].

### 2.4. Data Analysis

All the experiment was designed according to CRD (completely random design) and the data was analyzed using ANOVA significant differentiations compared by DMRT (Duncan Multiple Range Test).

## 3. Result & Discussion

### 3.1. Determination of Developmental Stage of Pollen Grain

Flower buds of different sizes were collected and evaluated, and the best sizes of the flower buds were about 6–8 mm long (Table 1). The anthers containing mid- or late-uninucleate microspores were most suitable for anther culture (Figure 2(b)). The stage of pollen grain factor is very important and seriously affects the success of anther culture. Generally, the period of sensitivity to inductive treatments is around the first pollen mitosis, that is, between the vacuolate microspore to early, mid-bicellular pollen [20]. It is probably due to the transcriptional status that at that time it is still proliferative and not yet fully differentiated [21]. The buds of less than 6 mm long before starting of meiosis stage for pollen mother cell (PMC) had white color for anther and stigma (Figure 2(a)), and buds of length 10, 15, and 20 mm (Figure 2(d)) have mature pollen grain (after pollen mitosis1) with different color and length anthers (Table 1, Figure 1). After the pollen grains begin to accumulate storage reserves, they usually lose their embryogenic capacity and follow the gametophytic developmental pathway [22]. In the present study, for the cooling pretreatment, there were no significant differences between chilling treatment and control treatment (direct culture after sterilized treatment). Ali [23] reported that some of crops were not affected by cool pretreatment. The optimum temperature and duration of pretreatment vary with the genotype.

### 3.2. Flower Initiation Time and Anther Collection

The higher percentage (90%) of callus formation was from anthers collected from plants which started of flowering (after 1-2 weeks), and after 4 weeks, the callus formation was decreased to 60% and after 7 weeks it was reduced to 35% (Table 2), Seguí-Simarro and Nuez [24] also report the same results on tomato.

### 3.3. Callus Induction

Callus started to appear after 2–4 weeks of incubation in the dark place. High frequency of callus formation was observed from T4 medium which contained NAA 3.0 mg/L and BAP 3.0 mg/L, while less frequency of callus formation was observed in media containing 2,4-D 2.0 mg/L and Kin 1.0 mg/L (Table 3 & Figure 2(g)). BAP and NAA have been widely used for indirect organogenesis in various protocols developed for the member of Malvaceae family [25, 26] and in other plants [27, 28]. After two weeks of incubation in dark, the callus has been subcultured to the fresh media containing 0.5 mg/L TDZ with 0.5 mg/L NAA and incubated in the light condition (Figure 2(h)). In okra, the morphogenic callus induction was observed in highest frequency from hypocotyl explants by culturing in MS medium supplemented with 2.0 mg/L NAA plus 0.5 mg/L TDZ [29].

## 4. Conclusion

The present study concludes that the suitable microspore stage of the kenaf flower buds was about 6–8 mm long, and the anther collected from plants at the beginning of flower initiation was induced higher frequency of callus formation. The best hormone combination for callus induction was found to beMS medium containing NAA 3.0 mg/L and BAP 2.0 mg/L. The protocols developed under this study are valuable to induce callogenesis from kenaf anthers, which may help in the production of haploid plant in kenaf.

## Figures and Tables

**Table 1:** Relation between flower bud length, anther size, and stigma color.

Bud length (mm)	Diameter of anther bunch (mm)	Anther length (mm)	Anther color	Stigma color
<6	<2.0	<0.4	White	Yellow
7.0 ± 1	3.7 ± 0.1	0.6 ± 0.2	White	Pink
10.0 ± 2	4.4 ± 0.6	0.9 ± 0.1	Yellow	Red
14.0 ± 2	5.2 ± 0.2	1.1 ± 0.1	Brown	Red
18 ± 2	>5.4	1.4 ± 0.2	Carmine	Red

**Table 2:** Frequency of callus formation of flower buds collected in different time after bud initiation.

Days after flower beginning	Frequency of callus formations (%)
1-2 weeks	90
4 weeks	60
7 weeks	35

**Table 3:** Effect of the different types of PGR on callus induction.

Number of treat	NAA mg/L	2,4-D mg/L	KIN mg/L	6-BA mg/L	Number of anthers inoculated	Average number of anthers responded	Callus formation frequency %
T1	2.0		1.0	—	60	3.0^de^	30
T2	2.0		2.0	—	60	2.5^ef^	25
T3	3.0		1.0	—	60	4.5^cd^	45
T4	3.0		2.0	—	60	5.0^c^	50
T5	2.0		—	2.0	60	7.3^ab^	73
T6	2.0		—	3.0	60	6.2^bc^	62
T7	3.0		—	2.0	60	8.0^a^	80
T8	3.0		—	3.0	60	9.0^a^	90
T9		1.0	1.0	—	60	2.0^ef^	20
T10		1.0	2.0	—	60	1.3^ef^	13
T11		2.0	1.0	—	60	1.0^f^	10
T12		2.0	2.0	—	60	3.0^de^	30
T13		1.0	—	2.0	60	2.5^ef^	25
T14		1.0	—	3.0	60	2.2^ef^	22
T15		2.0	—	2.0	60	3.0^de^	30
T16		2.0	—	3.0	60	2.5^ef^	25

Values followed by the same letters within a column are not significantly different (*P* ≤ 0.05); Duncan's Multiple Range Test (DMRT).
